# Friendship and Loneliness in Dementia Caregiving: Daily Experiences of High and Low Burden Caregivers

**DOI:** 10.1093/geroni/igaf122.1759

**Published:** 2025-12-31

**Authors:** Yee To (Crystal) Ng, Angela Turkelson, Anna Kratz, Kira Birditt

**Affiliations:** University of Michigan, Ann Arbor, Michigan, United States; University of Michigan Institute for Social Research, Ann Arbor, Michigan, United States; University of Michigan, Ann Arbor, Michigan, United States; University of Michigan, Ann Arbor, Michigan, United States

## Abstract

Dementia caregivers are particularly vulnerable to loneliness compared to non-dementia caregivers and non-caregivers. Grounded in the Social Convoy and Stress Process Models, this study examined whether (a) dementia caregivers with fewer close friends experience greater loneliness; (b) daily interactions with friends are linked to reduced momentary loneliness, and if the quality and closeness of these interactions matter; and (c) whether such links vary by caregiver burden. The sample included 223 dementia caregivers from the Stress and Well-Being in the Everyday Lives of Caregivers Study. They completed a baseline interview assessing their demographics, caregiving, and social network characteristics, followed by a five-day ecological momentary assessment (EMA), reporting their social interactions and loneliness every three hours. Findings revealed that greater number of close friends was associated with reduced momentary loneliness compared to those with fewer close friends in social networks. Caregivers reported interactions with friends in 22% of EMA surveys. Multilevel linear models showed that caregivers experienced lower loneliness during interactions with friends—especially positive interactions and with non-close friends—compared to when they did not interact with friends. Furthermore, friend interactions were associated with lower momentary loneliness for caregivers with higher burden but not lower burden. Findings suggest that caregivers with fewer friends experience more loneliness, and daily interaction with friends helps reduce momentary loneliness, particularly for high-burden caregivers. These results highlight the need for interventions that promote friendship connections, to alleviate loneliness and enhance dementia caregiver well-being.

